# Noise Pareidolia Test in Parkinson’s Disease and Atypical Parkinsonian Syndromes: A Retrospective Study

**DOI:** 10.7759/cureus.55436

**Published:** 2024-03-03

**Authors:** Takuya Matsumoto, Jinsoo Koh, Mayumi Sakata, Yoshiaki Nakayama, Shoko Yorozu, Junko Taruya, Maiko Takahashi, Katsuichi Miyamoto, Hidefumi Ito

**Affiliations:** 1 Department of Neurology, Wakayama Medical University, Wakayama, JPN

**Keywords:** noise pareidolia test, pareidolia, visual misperception, visual hallucination, atypical parkinsonian syndrome, parkinson’s disease

## Abstract

Introduction: Pareidolias, or visual misperceptions, are a non-motor symptom of Parkinson’s disease (PD) with unclear pathophysiology. The noise pareidolia test (NPT) is a tool for screening pareidolias. The usefulness of the NPT in differentiating PD from atypical parkinsonian syndromes (APS) is also unknown.

Methods: We retrospectively investigated 74 patients with PD and 18 patients with APS who took the NPT. Correlations between the number of pareidolic responses, gray matter volume, and cerebral blood flow were also examined in the patients with PD.

Results: The median number of pareidolic responses in patients with PD and patients with APS was 0 (interquartile range (IQR): 0-3) and 0 (IQR: 0-1), respectively, and tended to be higher in patients with PD than in those with APS (p = 0.077). It was significantly higher in patients with PD who had hallucinations (2; IQR: 0-9) (p = 0.016). The area under the receiver operating characteristic curve for the number of pareidolic responses in the NPT was 0.62 when used to differentiate PD and APS, and the optimal cutoff number of pareidolic responses was 2/3. Sensitivity and specificity were 25.7% and 100%, respectively. In the PD group, the number of pareidolic responses was correlated with age (r = 0.27; p = 0.021) and the Frontal Assessment Battery (FAB) score (r = −0.34; p = 0.0099). Magnetic resonance imaging showed no significant correlation between the number of pareidolic responses and the volume of focal gray matter. On cerebral hypoperfusion mapping, the left parietal lobe had a significant correlation with the number of pareidolic responses (r = 0.35; p = 0.027).

Conclusion: The number of pareidolic responses in NPT was suggested to be useful as a red flag to rule out APS in differentiating PD from APS. In PD without dementia, the number of pareidolic responses was associated with reduced blood flow in the left parietal lobe.

## Introduction

Parkinson’s disease (PD) is a common neurodegenerative disease in which patients suffer from motor symptoms and various non-motor ones [1]. Visual misperception is a non-motor symptom of PD, categorized as minor hallucinations [2], and has been reported to be present in 19%-25% of cases of PD without dementia [3], 58%-65% of cases of PD with dementia [3], and 32% of cases of dementia with Lewy bodies (DLB) [4]. Pareidolias are visual misperceptions in which ambiguous shapes are perceived as meaningful objects; they are considered to be a phenomenon similar to visual hallucinations [5]. Few tools are currently available for the assessment of pareidolias, or visual hallucinations. The scenery pareidolia test [6] is a useful tool to detect pareidolias in PD but is time-consuming and thus unsuitable for routine clinical use. A simplified version, the noise pareidolia test (NPT), was subsequently developed and is now widely used in Japan. In this test, patients with DLB reportedly have pareidolias more frequently than patients with Alzheimer’s disease and more than healthy controls. Moreover, pareidolic responses in patients with DLB had a significant correlation with the severity of visual hallucinations, suggesting that NPT is a useful tool for the assessment of visual hallucinations in patients with DLB [7]. Patients with PD also reportedly have more pareidolic responses according to the NPT than those with multiple system atrophy (MSA) [8]. However, the influence of cognitive decline on pareidolic responses could not be excluded from that study because it included patients with dementia.

Furthermore, the pathophysiology remains unclear. Recent studies have suggested that pareidolias are associated with hypometabolism in the parietal, temporal, and occipital lobes [6], decreased cerebral blood flow in the bilateral frontal lobes, left cingulate cortex, and left angular and superior marginal gyri [9], decreased dopamine transporter uptake in the right striatum [10], altered frontotemporal connectivity [11], disturbed interactions between the dorsal and ventral attention networks [12], and increased normalized clustering coefficient and lower frontal degree centrality examined using electroencephalogram [13]. However, evidence of its pathophysiology is inadequate.

In this study, we investigated the usefulness of NPT in non-demented PD and atypical parkinsonian syndromes (APS), including MSA with predominant parkinsonism (MSA-P) and progressive supranuclear palsy (PSP). We also examined the relationship between the result of the NPT and cerebral blood flow assessed using ^99^ ^m^Tc-ethyl cysteinate dimer (ECD)-single-photon emission computed tomography (SPECT).

## Materials and methods

Study design

The results of the NPT in non-demented patients with PD, MSA, and PSP were retrospectively examined to determine its characteristics and diagnostic ability. Additionally, we examined the association between NPT scores with brain MRI and cerebral perfusion SPECT in the group of patients with PD.

Setting

This study was conducted at a single institution, Wakayama Medical University, a university hospital in Wakayama, Japan. Data from the subjects were consecutively collected between January 2015 and December 2020. Inpatients with PD, MSA-P, and PSP who were admitted for diagnosis, drug adjustment, and consideration of indications for device therapy during the period of interest and who underwent the NPT were retrospectively identified from the database.

Subjects

The eligibility criteria were as follows: patients with PD, MSA-P, and PSP whose Mini-Mental State Examination (MMSE) scores were ≥24. Clinical diagnoses were made according to the United Kingdom Parkinson’s Disease Society Brain Bank criteria for PD [14], the criteria described in the second consensus statement by Gilman et al. for probable or possible MSA [15], and the Movement Disorder Society clinical diagnostic criteria for probable or possible PSP [16]. Excluded were patients with ophthalmologic diseases or visual field abnormalities and those with focal brain lesions or severe ischemic changes on imaging studies.

We collected patient characteristics, including age, sex, disease duration, levodopa equivalent daily dose (LEDD) [17], the Unified Parkinson’s Disease Rating Scale (UPDRS) part III, MMSE, Frontal Assessment Battery (FAB), and the presence of clinical visual hallucinations or misperceptions. The UPDRS part III scores were assessed while the patients were in the on state, i.e., when their motor symptoms were sufficiently improved by medication.

Noise pareidolia test

The NPT, which was developed at Tohoku University in Japan, is a simple neuropsychologic test to evoke and measure visual misperceptions [7]. In this test, the subjects are gradually presented with 40 task images. Each image is presented within 30 seconds. The task images consist of black-and-white patterns, and eight of the 40 images are embedded with images of human faces; 32 are not. The subjects were instructed to answer whether a task image contained a human face. If faces were perceived, the subjects were requested to answer “yes” and to point to the area where they thought these faces were, and if not, they were requested to answer “no.” They were instructed to answer “yes” only when faces were clearly seen after explanation and three training trials had been completed. Patient responses were categorized into three types: (1) ‘correct responses’, in which the subjects correctly identified the faces contained in the task images or correctly responded that there were no faces; (2) ‘pareidolic responses’, in which the subjects falsely identified faces that were not actually in the images; and (3) 'miss’, in which the subjects failed to detect the faces embedded in the task images. Subjects received no feedback, regardless of whether or not the responses were correct.

Magnetic resonance imaging acquisition and analysis

A 3-Tesla MRI scanner (Siemens MAGNETOM Skyra, Siemens Medical Solutions USA, Inc., Malvern, PA) with a 32-channel head coil was used to scan each subject. The following parameters were used to acquire T1-weighted structural images (sagittal): repetition time = 580 ms, echo time = 11.0 ms, field of view = 220 mm, base resolution = 256, and slice thickness = 0.55 mm.

To perform voxel-based morphometry, we used Statistical Parametric Mapping Version 12 (SPM12, Functional Imaging Laboratory, UCL Queen Square Institute of Neurology, London, UK) in MATLAB (The MathWorks, Inc., Natick, MA). The T1-weighted structural images were segmented into gray matter, white matter, cerebrospinal fluid, and other non-brain parts. Spatial normalization was performed on gray matter probability maps using the diffeomorphic anatomical registration through the exponentiated Lie algebra algorithm. Normalized images were smoothed using an 8-mm full width at half maximum Gaussian kernel. Age was included as a covariate. The total brain volume was applied for global calculations. The masking images were created using T1-weighted images of all subjects, and implicit and explicit masking were adapted. Multiple regression analysis was performed to evaluate the correlation between the number of pareidolic responses in the NPT and the volume of gray matter. Family-wise error-corrected p-values < 0.05 were considered significant, and uncorrected p-values < 0.001 were also analyzed for exploratory analysis. 

Acquisition of SPECT data and analysis

Images from ^99 m^Tc-ECD-SPECT were acquired using the Infinia Discovery NM630 (General Electric Co., Boston, MA), which was equipped with low-energy, high-resolution collimators. Before acquisition, the patients were placed in the supine position in a quiet room and were instructed to keep their eyes closed during imaging. The scan was started five minutes after an intravenous injection of 600 mBq of ^99 m^Tc-ECD. The SPECT images were continuously obtained using a 64 × 64 matrix of 4.42-mm pixel size over 360° in 4° steps for four rotations, with a zoom of 2.00. The total acquisition time was 18 minutes. The projection data were pre-filtered through a Butterworth filter (cutoff, 0.5 cycle/cm; power, 10) and reconstructed using a Ramp filter with the application of Chang’s attenuation correction (µ = 0.11 cm^−1^). Scatter correction was not performed.

Analysis of SPECT imaging was performed using SPM12, an easy Z-score imaging system (eZIS, Fujifilm RI Pharma, Tokyo, Japan), and voxel-based stereotactic extraction estimation (vbSEE, Fujifilm RI Pharma, Tokyo, Japan), as previously reported [18-20]. After anatomical normalization using SPM12, we used eZIS to investigate decreases in cerebral blood flow. The Z-scores were calculated for each patient according to the degree of decrease in regional ^99 m^Tc-ECD uptake compared with the normal database. The Z-score was defined as follows: {(normal database mean) − (individual value)}/(control standard deviation). Subsequently, we used vbSEE to convert the Z-score map data into a Talairach brain coordinating space. Voxel-based stereotactic extraction estimation was used to calculate the percentage of voxels that showed an uptake reduction of > 2 standard deviations from the average of controls in a given volume of interest (VOI), designated as extent. Voxel-based stereotactic extraction estimation can change the VOI to hemisphere, lobe, and lobule levels; in this study, we selected the lobe and lobule levels. The extent of the target VOIs was used to examine the correlation between the number of pareidolic responses and decreases in ^99 m^Tc-ECD uptake in the target VOIs.

Statistical analysis

All statistical analyses, except for the structural MRI data analysis, were performed using JMP Pro, version 14.1.0 (SAS Institute, Cary, NC). Demographic data of the patients with PD and APS were compared using the chi-square test for sex and visual hallucination or misperception and the Mann-Whitney U test for age, disease duration, LEDD, UPDRS part III, MMSE, and FAB. Correlations between the number of pareidolic responses and the clinical data of patients with PD were evaluated using Pearson’s correlation coefficient. Also, linear multiple regression analysis was performed using the stepwise method to identify the determinants of the number of pareidolic responses. To investigate the ability of the NPT to differentiate PD from APS, receiver operating characteristic curves were used, and the cutoff scores were defined using the Youden index [21]. Missing values were excluded from the statistical analyses. P-values < 0.05 were used to denote statistical significance.

## Results

We collected data on 74 patients with PD and 18 patients with APS (Table 1). In patients with PD, UPDRS part III and FAB score data were not available for two and 17 patients, respectively. No statistically significant differences in sex, age, disease duration, LEDD, or MMSE were observed between the two groups. The APS group had significantly lower FAB scores than the PD group (p = 0.026).

**Table 1:** Demographic and clinical profiles of patients with PD and APS Data are shown as absolute numbers or medians (interquartile ranges). ^a^ Chi-square test; ^b^ Mann–Whitney U test PD: Parkinson’s disease; APS: atypical parkinsonian syndrome; LEDD: levodopa equivalent daily dose; UPDRS: Unified Parkinson’s Disease Rating Scale; MMSE: Mini-Mental State Examination; FAB: Frontal Assessment Battery

Group	PD (n = 74)	APS (n = 18)	P-values
Sex, n (female/male)	41/33	12/6	0.39^a^
Age, year	67.5 (58–74)	65 (53–73.25)	0.36^b^
Disease duration, month	31 (15.75–98.5)	18.5 (13.5–75)	0.19^b^
Visual hallucination or misperception, n	11	0	0.081^a^
LEDD, mg/day	214.8 (0–985.9)	112.5 (0–828.4)	0.40^b^
UPDRS part III	19 (13–28)	−	−
MMSE	28 (26–30)	28 (25.75–29)	0.18^b^
FAB	15 (13–17)	14 (10.75–16)	0.027^b^

**Figure 1:**
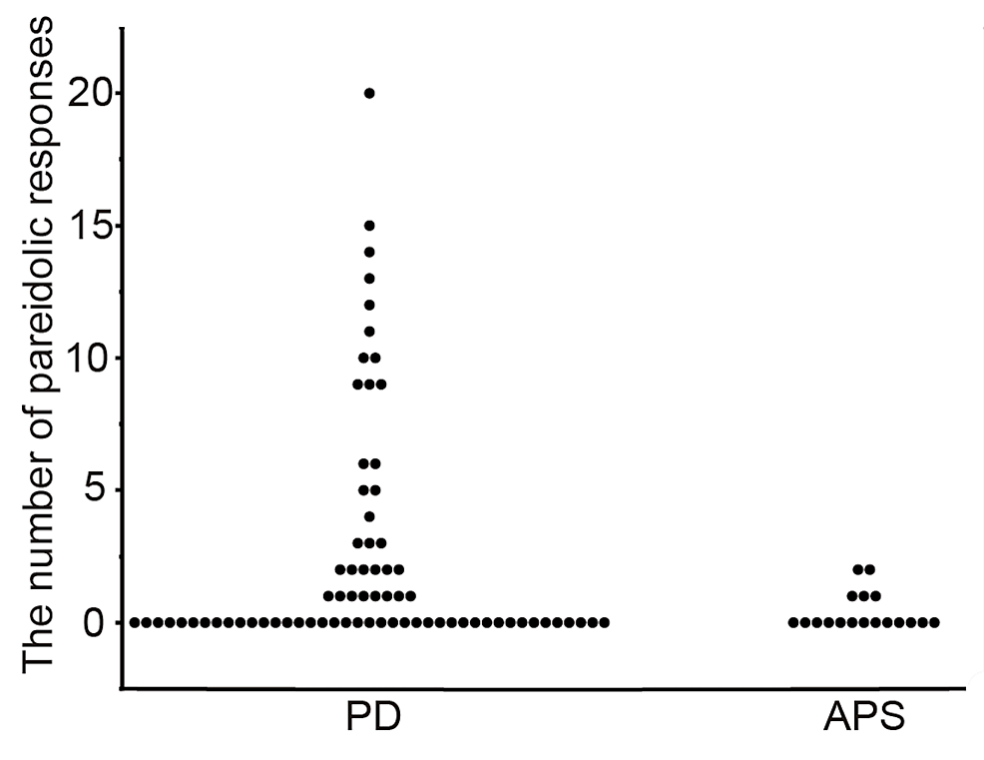
Number of pareidolic responses on the noise pareidolia test in patients with PD and APS The number of pareidolic responses tended to be higher (p = 0.077) and had a larger variance in PD than in APS. PD: Parkinson’s disease; APS: atypical parkinsonian syndrome

**Figure 2:**
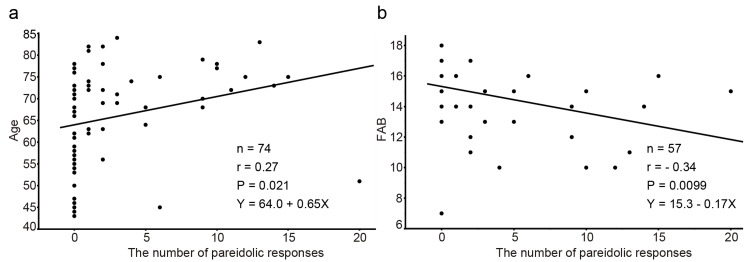
Correlation of age and FAB score with pareidolia in patients with PD The pareidolic responses correlated with (a) age (r = 0.27; p = 0.021) and (b) FAB score (r = −0.34; p = 0.0099). FAB: Frontal Assessment Battery; PD: Parkinson’s disease

Regarding the pareidolic responses assessed using the NPT in PD and APS, the median number of pareidolic responses was 0 (interquartile range (IQR): 0-3) and 0 (IQR: 0-1), respectively; the mean number of pareidolic responses was 2.5 ± 4.4 (95% confidence intervals (95% CI), 1.5-3.5) and 0.4 ± 0.7 (95% CI, 0.0-0.7), respectively; the maximum number of pareidolic responses was 20 and two, respectively (Figure 1). The pareidolic responses tended to be higher in PD than in APS (p = 0.077). In 11 patients with PD who had experienced hallucinations, the number of pareidolic responses was two (IQR: 0-9), which was significantly higher than that in patients with APS (p = 0.016); conversely, this number was not significantly different from that in patients with PD without hallucinations (median: 0; IQR: 0-2) (p = 0.15). The area under the curve for the pareidolic responses assessed using the NPT was 0.62 when used to differentiate patients with PD from those with APS. When the optimal cutoff for the pareidolic responses was 2/3, the sensitivity and specificity were 25.7% and 100%, respectively. In the group of patients with PD, patients with pareidolic responses (the number of pareidolic responses was ≥1) were significantly older and had lower FAB scores than those without pareidolic responses (Table 2). The pareidolic responses correlated with age (r = 0.27; p = 0.021) and FAB score (r = −0.34; p = 0.0099) (Figure 2) but did not correlate with disease duration (r = 0.14; p = 0.24), LEDD (r = 0.15; p = 0.19), UPDRS part 3 (r = 0.094; p = 0.43), or MMSE (r = −0.15; p = 0.19).

**Table 2:** Demographic and clinical profiles of PD patients with and without pareidolic responses Data are shown as absolute numbers or medians (interquartile ranges). ^a^ Chi-square test; ^b^ Mann–Whitney U test PD: Parkinson’s disease; APS: atypical parkinsonian syndrome; LEDD: levodopa equivalent daily dose; UPDRS: Unified Parkinson’s Disease Rating Scale; MMSE: Mini-Mental State Examination; FAB: Frontal Assessment Battery

Group	PD with pareidolic responses (n = 33)	PD without pareidolic responses (n = 41)	P-values
Sex, n (female/male)	15/18	26/15	0.12^a^
Age, year	72 (66–77.5)	61 (55.5–70.5)	< 0.001^b^
Disease duration, month	37 (16.5–126)	29 (15.5–96.5)	0.61^b^
Visual hallucination or misperception, n	7	4	0.17^a^
LEDD, mg/day	300 (0–1067)	0 (0–933)	0.17^b^
UPDRS part III	21 (13–28)	18 (12–26.5)	0.46^b^
MMSE	28 (27–29)	29 (26–30)	0.17^b^
FAB	14 (12–16)	16 (15–17)	0.0009^b^

Structural MRI data were available for all 74 patients with PD. The multiple regression analysis on MRI images showed no significant correlations between the number of pareidolic responses and regional gray matter volume. However, the number of pareidolic responses had a weak correlation with the bilateral angular and postcentral gyri, left frontal lobe (frontal eye field), and right frontal lobe (inferior frontal gyrus) (uncorrected, p < 0.001).

**Figure 3:**
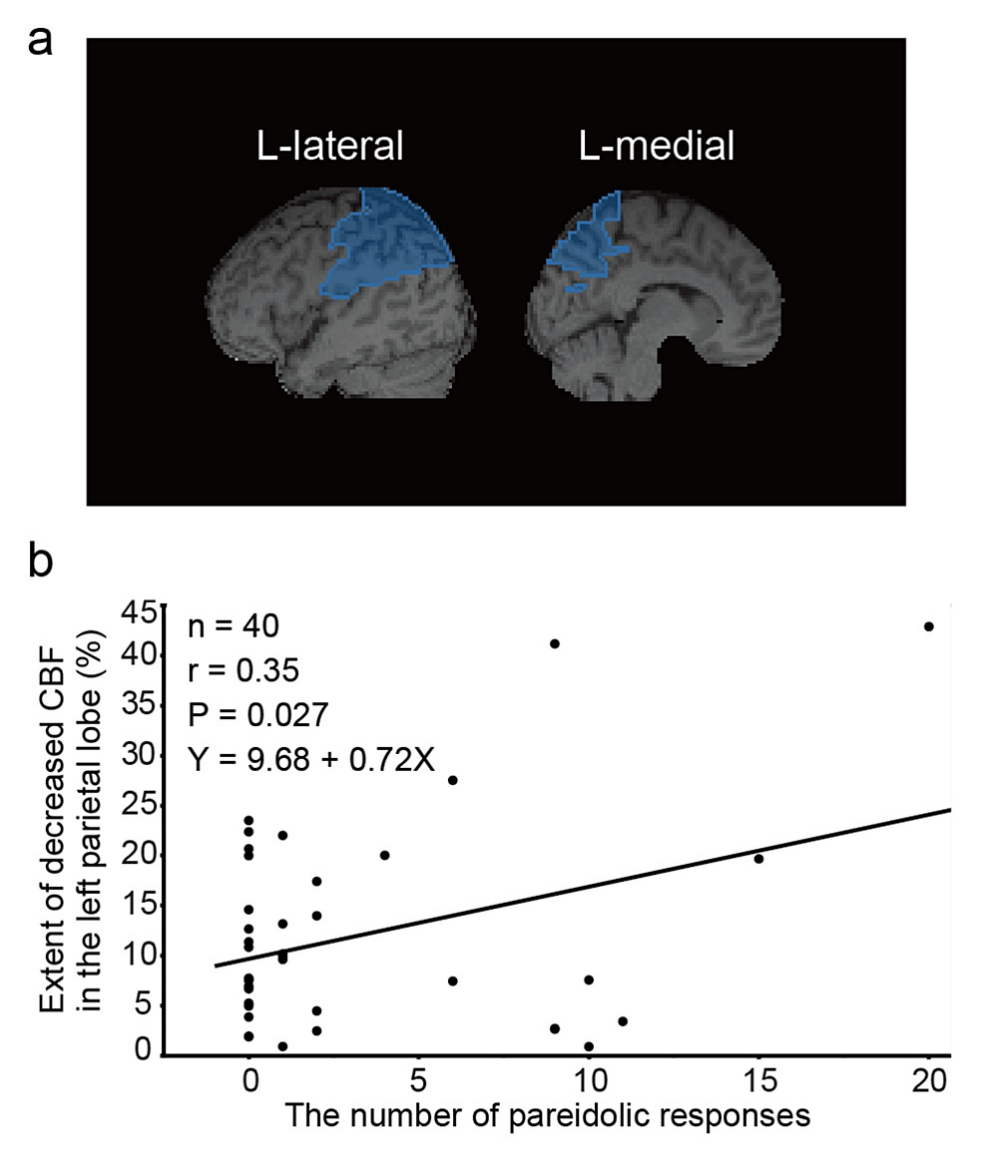
Correlation between pareidolia and left parietal lobe cerebral blood flow in patients with PD a: Brain surface image on vbSEE; the left parietal lobe is highlighted according to Talairach’s classification; b: The scatter plots show the extent of decrease in cerebral blood flow in the left parietal lobe and the number of pareidolic responses. PD: Parkinson’s disease; vbSEE: voxel-based stereotactic extraction estimation; L: left

The data of ^99 m^Tc-ECD SPECT were available for 40 patients with PD, whose demographics were not statistically different from those of all 74 patients with PD. On cerebral hypoperfusion mapping, the left parietal lobe had a significant correlation with the number of pareidolic responses (r = 0.35; p = 0.027) (Table 3 and Figure 3). Based on the aforementioned results, we applied a multiple regression model to the number of pareidolic responses as the dependent variable and with the following factors as independent variables: age, LEDD, FAB, disease duration, and extent of decreased cerebral blood flow in the left parietal lobe. Only the left parietal blood flow was identified as a significant independent variable (β = 0.17; p = 0.027).

**Table 3:** Correlations between pareidolia and the extent of decreased cerebral blood flow *p-values < 0.05

		r	P-values
Frontal lobe	Left	−0.042	0.80
	Right	−0.091	0.58
Parietal lobe	Left	0.35	0.027*
	Right	0.21	0.18
Postcentral gyrus	Left	0.38	0.017*
	Right	0.23	0.16
Superior parietal lobule	Left	0.33	0.040*
	Right	0.21	0.20
Inferior parietal lobule	Left	0.38	0.016*
	Right	0.24	0.13
Precuneus	Left	0.16	0.32
	Right	0.06	0.71
Temporal lobe	Left	−0.056	0.73
	Right	−0.029	0.86
Occipital lobe	Left	0.077	0.64
	Right	0.10	0.54

## Discussion

We investigated the usefulness of the NPT in differentiating PD from APS. The number of pareidolic responses tended to be higher in PD than in APS and had a large variance in the PD group, which reflected the heterogeneity in PD. Conversely, almost no pareidolic responses were observed in non-demented patients with APS. Pareidolic responses are thus suggested to be a specific symptom of Lewy body disease. Furthermore, the number of pareidolic responses was significantly higher in PD with visual hallucinations, suggesting that there are certain pathophysiological commonalities between visual hallucinations and pareidolic responses.

The NPT is considered unsuitable as a diagnostic marker of PD because of its low sensitivity. However, a high number of pareidolic responses can exclude APS because it has high specificity. Our data suggested a cutoff value of 2/3, although the data were not robust because of the small sample size. Indeed, in one report, some patients with MSA showed more than three pareidolic responses [8]. However, patients with cognitive impairment were not excluded from this study, which could have influenced the results of the NPT. Nevertheless, the number of pareidolic responses in MSA never exceeded four. Pareidolias might, therefore, be a red flag for PD.

The number of pareidolic responses had a weak correlation with age and the FAB score. A previous study on the scenery pareidolia test in non-demented patients with PD also suggested a correlation between the number of pareidolic responses and both age and FAB scores [6]. Pareidolias may, therefore, be associated with age and the FAB score, regardless of the testing method. Conversely, the APS group with fewer pareidolic responses had lower FAB scores. Pareidolia is, therefore, not thought to be caused by the frontal dysfunction itself but by the neuropsychiatric dysfunction associated with PD pathology in common with a decrease in the FAB.

We suggested that pareidolic responses were correlated with left parietal lobe hypoperfusion, but were not associated with frontal or occipital lobe hypoperfusion. This result was also reproduced in the multivariate analysis, but pareidolic responses tended to be correlated with frontal and parietal atrophy. A report also showed that hypometabolism in the left parietal lobe was associated with scenery pareidolias and visual hallucinations [6]. Furthermore, in a study on DLB, NPT scores were weakly correlated with decreased regional cerebral blood flow in the bilateral frontal lobes, left cingulate cortex, and left angular and superior marginal gyri [9]. The mechanism of visual hallucinations in Lewy body disease remains under debate; one hypothesis attributes this symptom to dysfunction of the attentional control networks. This model suggests that visual misperceptions and hallucinations are caused by dysfunction of the dorsal attention network, which consists of the frontal eye field, the dorsolateral prefrontal cortex, and the superior posterior parietal cortex [12]. Pareidolias may therefore be associated with abnormal attentional control networks involving the parietal and frontal lobes.

This study has some limitations. First, the patients were tested while continuing their usual medications, so the influence of dopaminergic therapy cannot be ignored. The pareidolic responses addressed in this study were to be deemed a mixture of drug-induced effects and PD pathology itself. Indeed, LEDDs were slightly higher in PD with pareidolia than in PD without pareidolia, but without statistical significance. Pareidolia in drug-naïve PD was, however, shown in one previous study [10]. Moreover, pareidolias were shown in multivariate analysis to be associated with left parietal hypoperfusion, but not LEDDs. We therefore speculated that pareidolias were more related to the pathophysiology of PD itself than to anti-parkinsonian drugs. A second limitation of this study is the possibility that the number of patients in the APS group was too small for the detection of differences from the PD group. Third, this was a retrospective study, and data on motor and non-motor symptoms were not fully available. Although the relationship between pareidolias and motor symptoms remains controversial [10,13], this study showed no correlation between pareidolias and UPDRS part III scores.

## Conclusions

The number of pareidolic responses assessed using the NPT showed high variance in PD; however, this number was significantly higher in PD with visual hallucinations than in APS. In APS, the number of pareidolic responses was ≤ 2 and this may be used as a red flag to rule out APS in differentiating PD from APS. Furthermore, pareidolias in PD without dementia were suggested to be associated with reduced blood flow in the left parietal lobe.

## References

[1] Barone P, Antonini A, Colosimo C (2009). The PRIAMO study: a multicenter assessment of nonmotor symptoms and their impact on quality of life in Parkinson's disease. Mov Disord.

[2] Fénelon G, Mahieux F, Huon R, Ziégler M (2000). Hallucinations in Parkinson's disease: prevalence, phenomenology and risk factors. Brain.

[3] Archibald NK, Clarke MP, Mosimann UP, Burn DJ (2011). Visual symptoms in Parkinson's disease and Parkinson's disease dementia. Mov Disord.

[4] Stavitsky K, Brickman AM, Scarmeas N (2006). The progression of cognition, psychiatric symptoms, and functional abilities in dementia with Lewy bodies and Alzheimer disease. Arch Neurol.

[5] Uchiyama M, Nishio Y, Yokoi K, Hirayama K, Imamura T, Shimomura T, Mori E (2012). Pareidolias: complex visual illusions in dementia with Lewy bodies. Brain.

[6] Uchiyama M, Nishio Y, Yokoi K, Hosokai Y, Takeda A, Mori E (2015). Pareidolia in Parkinson's disease without dementia: a positron emission tomography study. Parkinsonism Relat Disord.

[7] Yokoi K, Nishio Y, Uchiyama M, Shimomura T, Iizuka O, Mori E (2014). Hallucinators find meaning in noises: pareidolic illusions in dementia with Lewy bodies. Neuropsychologia.

[8] Kurumada K, Sugiyama A, Hirano S (2021). Pareidolia in Parkinson's disease and multiple system atrophy. Parkinsons Dis.

[9] Nakata T, Shimada K, Iba A (2022). Correlation between noise pareidolia test scores for visual hallucinations and regional cerebral blood flow in dementia with Lewy bodies. Ann Nucl Med.

[10] Murakami H, Shiraishi T, Umehara T (2021). Face pareidolia is associated with right striatal dysfunction in drug-naïve patients with Parkinson's disease. Neurol Sci.

[11] Kajiyama Y, Hattori N, Nakano T (2021). Decreased frontotemporal connectivity in patients with parkinson's disease experiencing face pareidolia. NPJ Parkinsons Dis.

[12] Göbel N, Möller JC, Hollenstein N (2021). Face perception and pareidolia production in patients with Parkinson's disease. Front Neurol.

[13] Revankar GS, Kajiyama Y, Hattori N (2021). Prestimulus low-alpha frontal networks are associated with pareidolias in Parkinson's disease. Brain Connect.

[14] Hughes AJ, Daniel SE, Kilford L, Lees AJ (1992). Accuracy of clinical diagnosis of idiopathic Parkinson's disease: a clinico-pathological study of 100 cases. J Neurol Neurosurg Psychiatry.

[15] Gilman S, Wenning GK, Low PA (2008). Second consensus statement on the diagnosis of multiple system atrophy. Neurology.

[16] Höglinger GU, Respondek G, Stamelou M (2017). Clinical diagnosis of progressive supranuclear palsy: the Movement Disorder Society criteria. Mov Disord.

[17] Tomlinson CL, Stowe R, Patel S, Rick C, Gray R, Clarke CE (2010). Systematic review of levodopa dose equivalency reporting in Parkinson's disease. Mov Disord.

[18] Waragai M, Yamada T, Matsuda H (2007). Evaluation of brain perfusion SPECT using an easy Z-score imaging system (eZIS) as an adjunct to early-diagnosis of neurodegenerative diseases. J Neurol Sci.

[19] Uruma G, Hashimoto K, Abo M (2013). A new method for evaluation of mild traumatic brain injury with neuropsychological impairment using statistical imaging analysis for Tc-ECD SPECT. Ann Nucl Med.

[20] Mizumura S, Kumita S, Cho K, Ishihara M, Nakajo H, Toba M, Kumazaki T (2003). Development of quantitative analysis method for stereotactic brain image: assessment of reduced accumulation in extent and severity using anatomical segmentation. Ann Nucl Med.

[21] Youden WJ (1950). Index for rating diagnostic tests. Cancer.

